# Association between subjective oral dysfunction and locomotive syndrome in community-dwelling older adults

**DOI:** 10.1038/s41598-021-92153-8

**Published:** 2021-06-15

**Authors:** Misa Nakamura, Masakazu Imaoka, Hidetoshi Nakao, Mitsumasa Hida, Fumie Tazaki, Ryota Imai, Hirotoshi Utsunomiya, Hiroshi Hashizume

**Affiliations:** 1 Cognitive Reserve Research Center, Osaka Kawasaki Rehabilitation University, Kaizuka, Osaka Japan; 2 Department of Rehabilitation, Osaka Kawasaki Rehabilitation University, Kaizuka, Osaka Japan; 3 Department of Strategic Surveillance for Functional Food andComprehensive Traditional Medicine, Wakayama Medical University, Wakayama, Wakayama Japan; 4 Department of Orthopaedic Surgery, Wakayama Medical University, Wakayama, Wakayama Japan; 5 School of Health and Nursing Science, Wakayama Medical University, Wakayama, Wakayama Japan

**Keywords:** Health care, Dentistry, Geriatrics, Rheumatology, Musculoskeletal system

## Abstract

The need for support and care is a major problem facing societies around the world. Locomotive syndrome (LS) refers to a condition in which people require healthcare services because of problems associated with locomotion. Oral dysfunction is also associated with various long-term care factors including activities of daily living. The purpose of this study was to determine the association between oral dysfunction and LS. The study participants were 407 elderly people living in a rural area in Japan. Evaluation of oral dysfunction was based on subjective judgment by each participant. LS was assessed using Locomo-25, which is a self-administered questionnaire and was defined by a Locomo-25 score ≥ 7 points. Those with a “decline in masticatory function” and “difficulty swallowing” had higher odds of LS than those without these dysfunctions (odds ratio (OR) = 2.134, 2.007, respectively). Furthermore, participants with a Locomo-25 score ≥ 11 had higher odds of a “decline in masticatory function” (OR = 2.657) than those with a Locomo-25 score < 11, and those with a Locomo-25 score ≥ 9 had higher odds of “difficulty swallowing” (OR = 2.411) than those with a Locomo-25 score < 9. These findings suggest that a strong relationship exists between oral dysfunction and LS.

## Introduction

The need for support and care is a major problem facing societies around the world. Causes of need for support and care include falls, fractures, joint diseases, and other disorders that affect locomotion. A concept referred to as locomotive syndrome (LS) was proposed by the Japanese Orthopaedic Association. Risk increases as a person’s movement function deteriorates due to locomotive impairment. Some causes of LS are reduced muscle strength and balance associated with aging and conditions such as osteoporosis, osteoarthritis, and sarcopenia^1^. Motor functions such as grip strength and walking speed are also related to LS^2–5^.

At present, the concepts of LS, sarcopenia, and frailty have many things in common, and the distinction among them is ambiguous. The concept of LS includes social significance in which people’s understanding of locomotion leads to attention to motor disorders. The proportion of the Japanese population with LS (47 million) is estimated to be more than twice that with metabolic syndrome (20 million)^6^.

The World Health Organization is advocating for effective health promotion and intervention methods to improve oral health^7^. Oral frailty, which was proposed by the Japanese Ministry of Health, Labor and Welfare in 2013, is a deterioration of mouth function and eating function disorders that lead to mental and physical dysfunction due to neglect of minor deterioration of the mouth or improper treatment^8^. Recently, much attention has been focused on the relationship between oral health and sarcopenia^9^, or frailty^10^. The results of several large cohort studies showed a relationship between reduced masticatory function and increased risk of needing care and shortened life expectancy; decreased number of teeth and oral function and occurrence of physical dysfunction; and tooth loss and mortality^11–13^.

Therefore, the purpose of this study was to determine the association between LS and oral health, with the goal of providing information that will help prevent LS and oral dysfunction in community-dwelling elderly people.

## Results

### Characteristics of the study participants

Age, Locomo-25 score, Geriatric Depression Scale (GDS-15) score, Mini-Mental State Examination (MMSE) score, body mass index (BMI), LS status, and subjective oral function are shown in Table 1. The proportion of men was 22.85%. The average age was 73.13 ± 6.90 years, and 176 participants had LS (43.24%). In the LS risk classification, the proportion of participants with LS risk 1 was 27.27%, with LS risk 2 was 7.89%, and with LS risk 3 was 8.11%. The proportion of participants with “decline in masticatory function” was 23.59%, with “difficulty swallowing” in 26.29%, and with “Dry mouth” in 30.86%.

**Table 1:** Characteristics of the study participants.

Participants (male, %)	407 (22.85%)
Age (years)	73.13 ± 6.90
Locomo-25 score (points)	8.61 ± 9.88
GDS-15 score (points)	3.56 ± 2.73
MMSE score (points)	28.65 ± 2.14
BMI (kg/m^2^)	22.61 ± 3.09
**LS**	176 (43.24%)
LS risk 1	111 (27.27%)
LS risk 2	32 (7.86%)
LS risk 3	33 (8.11%)
**Oral dysfunction**	
Decline in masticatory function	96 (23.59%)
Difficulty swallowing	107 (26.29%)
Dry mouth	126 (30.86%)

Values are presented as means (standard deviation) or prevalence (%).

LS risk 1, Locomo 25 total score of 7–15 points; LS risk 2, 16–23 points; LS risk 3, 24 points or more.

*GDS-15* geriatric depression scale; *MMSE* Mini-Mental State Examination; *BMI* body mass index; *LS* locomotive syndrome.

### Odds ratios of characteristics for LS

Table 2 shows the odds ratios (ORs) of each measurement, including sex, age, GDS-15 scores, MMSE score, BMI, “decline in masticatory function”, “difficulty swallowing”, and “dry mouth” for LS on univariate and multiple logistic regression analyses. Univariate regression analysis showed that male sex (OR = 0.549, 95% confidence interval (CI) = 0.331–0.911; *p* = 0.0173), age (OR = 1.051, 95% CI = 1.020–1.083; *p* = 0.0012), GDS-15 score (OR = 1.275, 95% CI = 1.170–1.380; *p* < 0.0001), MMSE score (OR = 0.878, 95% CI = 0.796–0.969; *p* = 0.0094), BMI (OR = 1.105, 95% CI = 1.035–1.181; *p* = 0.0029), “decline in masticatory function” (OR = 2.954, 95% CI = 1.846–4.728; *p* < 0.0001), and “difficulty swallowing” (OR = 2.440, 95% CI = 1.555–3.828; *p* < 0.0001) were significantly associated with LS. Multiple regression analysis showed that male sex (OR = 0.450, 95% CI = 0.255–0.795; *p* = 0.0060), age (OR = 1.041, 95% CI = 1.005–1.078; *p* = 0.0270), GDS-15 scores (OR = 1.263, 95% CI = 1.156–1.381; *p* < 0.0001), MMSE score (OR = 0.926, 95% CI = 0.826–1.039; *p* = 0.1898), BMI (OR = 1.116, 95% CI = 1.037–1.202; *p* = 0.0036), “decline in masticatory function” (OR = 2.134, 95% CI = 1.270–3.585; *p* = 0.0042), and “difficulty swallowing” (OR = 2.007, 95% CI = 1.214–3.317; *p* = 0.0066) were significantly associated with LS.

**Table 2:** Odds ratios of characteristics for LS.

	Univariate			Multiple		
	OR	95% CI	*p*	OR	95% CI	*P*
Sex (male)	0.549	0.331–0.911	0.0173	0.450	0.255–0.795	0.0060
Age	1.051	1.020–1.083	0.0012	1.041	1.005–1.078	0.0270
GDS-15	1.275	1.170–1.380	< 0.0001	1.263	1.156–1.381	< 0.0001
MMSE	0.878	0.796–0.969	0.0094	0.926	0.826–1.039	0.1898
BMI	1.105	1.035–1.181	0.0029	1.116	1.037–1.202	0.0036
Decline in masticatory function	2.954	1.846–4.728	< 0.0001	2.134	1.270–3.585	0.0042
Difficulty swallowing	2.440	1.555–3.828	< 0.0001	2.007	1.214–3.317	0.0066
Dry mouth	1.381	0.900–2.118	0.1399			

Univariate and multiple logistic regression analyses were performed.

*LS* locomotive syndrome; *OR* odds ratio; *CI* confidence interval; *GDS-15* Geriatric Depression Scale; *MMSE* Mini-Mental State Examination; *BMI* body mass index.

### Threshold scores of Locomo-25 for “decline in masticatory function” and “difficulty swallowing”

The receiver operating characteristic curve (ROC) analysis of Locomo-25 scores showed a threshold score of 11 points for discriminating “decline in masticatory function” (area under the curve (AUC) = 0.668, sensitivity = 48.96%, specificity = 77.81%, *p* < 0.0001) and 9 points for discriminating “difficulty swallowing” (AUC = 0.640, sensitivity = 50.47%, specificity = 71.67%, *p* = 0.0004) (Table 3).

**Table 3:** Threshold score of Locomo-25 for “decline in masticatory function” and “difficulty swallowing”.

	Locomo-25 score (points)	AUC	Sensitivity	Specificity	*p*
Decline in masticatory function	11	0.668	48.96	77.81	< 0.0001
Difficulty swallowing	9	0.640	50.47	71.67	0.0004

Receiver operating characteristic curve analysis was performed.

*AUC* area under the curve.

### Odds ratios of variables for “decline in masticatory function” and “difficulty swallowing” according to the Locomo-25 score

Univariate logistic regression analysis showed that participants with a Locomo-25 score ≥ 11 had an OR of 3.364 for a “decline in masticatory function” (95% CI = 2.079–5.444; *p* < 0.0001), and those with a Locomo-25 score ≥ 9 had an OR of 2.577 for “difficulty swallowing” (95% CI = 1.636–4.060; *p* < 0.0001). Multiple logistic regression analysis showed that participants with a Locomo-25 score ≥ 11 had an OR of 2.657 for a “decline in masticatory function” (95% CI = 1.578–4.472; *p* = 0.0002), and those with a Locomo-25 score ≥ 9 had an OR of 2.411 for “difficulty swallowing” (95% CI = 1.495–3.886; *p* = 0.0003). On multiple logistic regression analysis, other characteristics showed no significant ORs (Table 4).

**Table 4:** Odds ratios of variables for “decline in masticatory function” and “difficulty swallowing” according to the Locomo-25 score.

		“Decline in masticatory function”							“Difficulty swallowing”					
		Univariate			Multiple				Univariate			Multiple		
		OR	95% CI	*p*	OR	95% CI	*p*		OR	95% CI	*p*	OR	95% CI	*p*
Sex	Male	0.665	0.371–1.194	0.1718				Male	0.900	0.528–1.533	0.6960			
Age		1.041	1.006–1.077	0.0213	1.053	0.985–1.062	0.2428		1.010	0.978–1.043	0.5394			
Locomo-25	≥ 11	3.364	2.079–5.444	< 0.0001	2.657	1.578–4.472	0.0002	≥ 9	2.577	1.636–4.060	< 0.0001	2.411	1.495–3.886	0.0003
GDS-15		1.142	1.054–1.239	0.0013	1.082	0.991–1.181	0.0798		1.089	1.006–1.178	0.0343	1.707	0.526–5.535	0.3748
MMSE		0.901	0.816–0.995	0.0401	0.969	0.867–1.083	0.5800		0.964	0.873–1.065	0.4724			
BMI		1.023	0.950–1.101	0.5494					1.061	0.989–1.139	0.1001			

Univariate and multiple logistic regression analyses were performed.

*OR* odds ratio; *CI* confidence interval; *GDS-15* Geriatric Depression Scale; *MMSE* Mini-Mental State Examination; *BMI* body mass index.

### Odds ratios of each Locomo 25 item for “decline in masticatory function”, “difficulty swallowing”, and “dry mouth”

**Figure 1:**
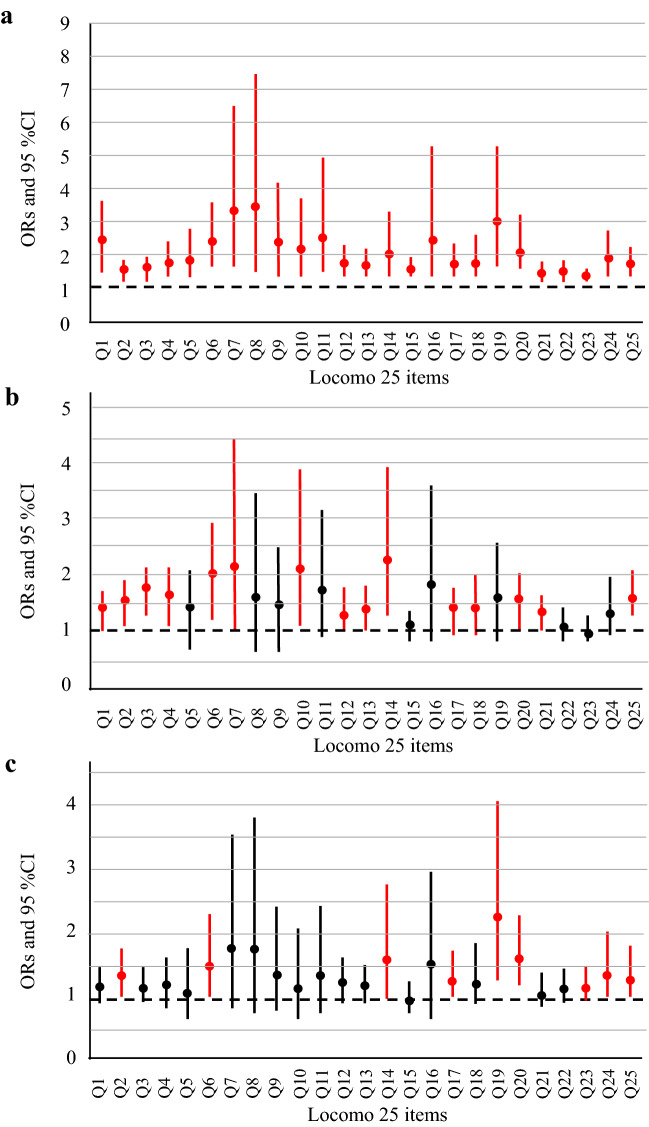
Odds ratios of Locomo 25 items for oral dysfunctions. Red color bars indicate a significant difference on univariate logistic regression analysis. (**a**) Odds ratios for “decline in masticatory function”. (**b**) Odds ratios for “difficulty swallowing”. (**c**) Odds ratios for “dry mouth”. OR, odds ratio; CI, confidence interval.

Figure 1 shows the ORs of each Locomo 25 item for “decline in masticatory function”, “difficulty swallowing”, and “dry mouth” on univariate logistic regression analysis. For “decline in masticatory function”, significant ORs were seen for all items (Fig. 1a). High ORs were seen especially for Q7 (“difficult to walk inside the house”, OR = 3.278, 95% CI = 1.573–6.831; *p* = 0.0015), Q8 (“difficult to put on and take off shirts”, OR = 3.439, 95% CI = 1.543–7.665; *p* = 0.0025), and Q19 (“difficult to do simple tasks and housework”, OR = 3.006, 95% CI = 1.694–5.333; *p* = 0.0002). For “difficulty swallowing”, significant ORs were seen for 15 items (Fig. 1b). High ORs were seen especially for Q7 (OR = 2.188, 95% CI = 1.076–4.451; *p* = 0.0307), Q10 (“difficult to use the toilet”, OR = 2.159, 95% CI = 1.186–3.933; *p* = 0.0119), and Q14 (“difficult to keep yourself neat”, OR = 2.308, 95% CI = 1.352–3.938; *p* = 0.0022). For “dry mouth”, significant ORs were seen for 9 items; an especially high OR was seen for Q19 (OR = 2.360, 95% CI = 1.351–4.125; *p* = 0.0026) (Fig. 1c).

## Discussion

This study showed a significant relationship between LS and decreased subjective oral function. Of the three subjective oral functions, masticatory function, swallowing function, and dryness of the mouth, a decline in masticatory function and difficulty swallowing were particularly associated with LS. Since the association with decreased masticatory function or dysphagia has not been investigated in the past, the threshold of the Locomo 25 score was also investigated in this new analysis. As a result of this ROC analysis, the Locomo-25 score was 11 points or more for a decline in masticatory function, and difficulty in swallowing function was significantly decreased at 9 points or more. Furthermore, a decrease in masticatory function showed a significant OR when the Locomo-25 score, which is an LS evaluation score, was 11 points or more (OR = 2.657), and difficulty in swallowing function showed a significant OR when the Locomo-25 score was 9 points or more (OR = 2.411).

Examining the associations of ORs and Locomo 25 items, significant ORs for “decline in masticatory function” were seen for all items, with high ORs for activities of daily living. This tendency was also seen for “difficulty swallowing”, and walking difficulty was particularly high for both oral dysfunctions.

Many reports have shown that motor functions such as grip strength, walking speed, and sarcopenia are related to LS^2–5^. On the other hand, masticatory function and tongue and lip function are related to physical functions such as grip strength^14,15^, gait speed^16^, the Timed-Up & Go Test, jump height^15^, life-space mobility^17,18^, and postural stability^19,20^. In addition, a relationship between LS and fall history has been reported^21^. Similarly, dysphagia is increasing in people with a history of falls^22^.

Furthermore, many reports of a relationship between oral health and sarcopenia have been published^9^. In the longitudinal studies, the number of teeth, subjective mastication, and tongue pressure, but not occlusal force, affected sarcopenia onset^7^. Maeda et al. reported that decreased swallowing function was correlated with the skeletal muscle mass index^23^.

A relationship is present between a decrease in motor function, which is a characteristic of LS, and oral function, which is a cause of poor nutrition. Poor oral health is a condition in which protein, calcium, vitamins, etc. related to the maintenance of locomotor function are deficient due to a decrease in the food intake level^24–26^. In addition, deterioration of nutritional status reduces the amount of physical activity and weakens locomotor function. Another possible cause is the involvement of the nervous system. Hatta et al. reported that peripheral orofacial sensory input may affect motor neuron control of muscle activity in other parts of the body^9^.

Locomo-25 includes not only structural and functional changes in locomotor function, but also mental states and connections with society. Reports have suggested a link between mastication and depression^27^ and that impaired social function may have a direct impact on impaired oral and physical function^28^. Deterioration of oral function is also expected to cause a decrease in conversational function, which will increase the risk of social deterioration and withdrawal. We infer that the mechanisms described above interact with each other to cause a decrease in oral function and an increase in LS.

Since a Locomo 25 total score of 7–15 points is LS risk 1 (the state in which movement function is starting to decline)^29^, masticatory function and swallowing function are considered to deteriorate from an early stage of LS, such as LS risk 1.

Dry mouth in males is significantly associated with incident falls^30^; however, few reports suggest a relationship between thirst and motor function. These results support the present study in which a decline in masticatory force and difficulty in swallowing function were associated with LS, but not between dry mouth and LS.

This study also investigated GDS-15 for evaluating depression and MMSE for evaluating cognitive function, which is related to oral health^31,32^ and LS^33,34^. It was confirmed that GDS-15 and MMSE are related to oral function and LS on univariate logistic regression analysis, as in the previous report. However, on multiple logistic regression analysis, the relationship between oral function and LS was the strongest.

This study has several limitations. First, only three types of tooth function items were investigated. In the future, investigation of objective indicators (number of teeth, tongue pressure, depth of the periodontal pocket on probing, mobility of dental elements, videoendoscopic examination of swallowing, and so on) will be necessary. Second, LS is evaluated not only by interviews with Locomo 25, but also by the stand-up test and the 2-step test, and the Japanese academic society of orthopedic surgery determines the locomotive degree based on the most severe stage of the locomotive degree based on the results of each test. In the future, it will be necessary to evaluate LS by including the results of these motor function tests. Third, data from a cross-sectional study are insufficient to determine whether a causal relationship exists between LS status and oral dysfunction. Therefore, conducting longitudinal studies to clarify the causal relationships among these factors is crucial. Fourth, selection bias cannot be ruled out, since healthy people could easily participate because the target persons were volunteers. Finally, since the results are based on the local residents of Osaka prefecture, the results could be different in different settings.

## Methods

### Participants

This study was conducted in a local area (Kaizuka City, Osaka Prefecture, Japan) between August 2018 and March 2019. The study inclusion criteria were: 1) Japanese, aged ≥ 55 years, and 2) living independently in their own home. All participants underwent weight and height measurements, followed by answering questionnaires at a public hall where a “Lecture meeting and checkup for health,” supported by the local government in Kaizuka, Osaka, was held. BMI was calculated as weight in kilograms divided by height in meters squared.

Of the 466 participants, 407 (mean age 73.13 ± 6.90 years; range 55–96 years) were analyzed, after excluding 13 participants under 55 years of age and 46 with incomplete data. All participants provided written, informed consent to being part of the research study. This study was conducted in accordance with the Declaration of Helsinki and was approved by the Ethics Committee of Osaka Kawasaki Rehabilitation University (Reference No OKRU28-A014).

### Assessment of LS status

LS status was evaluated using the Locomo-25 score (25-question Geriatric Locomotive Function Scale-25). The Locomo-25 is a self-administered questionnaire that comprises four questions about pain during the last month (Q1-Q4), 16 questions about activities of daily living during the last month (Q5-Q20), three questions about social function during the last month (Q21-Q23), and two questions about mental health status during the last month (Q24, Q25)^35^ (Supplementary Table S1). All 25 questions are scored from 0 (no impairment) to 4 (severe impairment), and the total score ranges from 0 to 100. Higher scores indicate worse locomotive function, and a total score of 7 points or more was judged as LS^29^. Furthermore, in the LS risk classification, a Locomo 25 total score of 7–15 points is LS risk 1, 16–23 points is LS risk 2, and 24 points or more is judged to be LS risk 3^29^. The validity of the Locomo-25 was confirmed by demonstrating a significant correlation with the European Quality of Life Scale-5 Dimensions questionnaire^35^.

### Assessment of oral dysfunction

Oral function was assessed using three items that evaluate oral function from the basic checklist developed for the purpose of evaluating the function of elderly people^36^ and predicting the risk of acquiring a need for nursing care. The basic checklist is a 25-item questionnaire and consists of items that evaluate instrumental activities of daily living, exercise, function, nutrition, oral function, withdrawal, cognitive function, and depression. Predictive validity for new events requiring long-term care has been reported for scales and subareas^37^. The questions were “Difficulties chewing tough foods (Decline in masticatory function)”, “Difficulties swallowing tea or soup (difficulty swallowing)”, and “experience having a dry mouth (dry mouth)”. The assessment of oral dysfunctions was based on the subjective judgment of each participant.

### Evaluation of cognitive function

Cognitive function was assessed using the MMSE, which assesses global cognition including orientation, registration, attention, calculation, language, and recall^38^.

### Evaluation of depression

Depression status was assessed using GDS-15, which is a commonly used instrument for depression screening in the general geriatric population. The GDS-15 is a yes/no questionnaire that does not focus on somatic symptoms and does not contain any questions about suicide. All 15 items are scored as either 0 or 1, and the total score ranges from 0 to 15. Scores from 5 to 9 indicate minor depressive disorder, and scores ≥ 10 indicate major depressive disorder^39^. The cut-off score for depression is 5 points; therefore, scores ≥ 5 were evaluated as depression.

### Statistical analysis

Participants were categorized into an LS group (Locomo-25 score ≥ 7) or a non-LS group (Locomo-25 score < 7). ORs of variables for LS status were calculated using univariate and multiple logistic regression analyses. Age, sex, GDS-15 score, MMSE score, BMI, decline in masticatory function, difficulty swallowing, and dry mouth were used as independent variables, and Locomo-25 ≥ 7 was considered the dependent variable. ORs of Locomo 25 items for oral dysfunction were calculated by univariate logistic regression analysis. The variance inflation factor was calculated using the least-squares method before performing the multiple logistic regression analysis to confirm that there was no multicollinearity between the independent variables. The Locomo-25 threshold score for discriminating the status of decline in masticatory function or difficulty swallowing was evaluated by ROC analysis. The ORs of Locomo-25 for the mouth status threshold score were calculated using univariate and multiple logistic regression analyses. Age, sex, threshold score of Locomo-25, GDS-15 score, MMSE score, and BMI were used as independent variables, and a decline in masticatory function or difficulty swallowing was considered the dependent variable. All *p* values and 95% CIs of two-sided analysis are presented. Statistical analysis was conducted using JMP 11 (SAS Institute, Cary, NC). All statistical tests were two-tailed, and a significance level of 0.05 was used.

## Supplementary Information

Supplementary Information.

## Data Availability

Data and materials can be obtained by contacting the corresponding author.
