# Supplementary material for: Association between subjective oral dysfunction and locomotive syndrome in community-dwelling older adults
Source: Sci Rep. 2021 Jun 15;11:12591. doi: 10.1038/s41598-021-92153-8 (PMC8206075; doi:10.1038/s41598-021-92153-8)
Supplement: Supplementary file 1 — Supplementary Information. [file 41598_2021_92153_MOESM1_ESM.docx]

Association between subjective oral dysfunction and locomotive syndrome in community-　dwelling older adults

Misa Nakamura, Masakazu Imaoka, Hidetoshi Nakao, Mitsumasa Hida, Fumie Tazaki, Ryota Imai, Hirotoshi Utsunomiya, Hiroshi Hashizume

**Supplementary Table S1.** The Locomo 25 questionnaire^35^

The following questions are asking about your health status and usual daily life, relating to the involvement of your back and limbs. Please answer about your status ‘over the last one month’.

| Following are questions about your body pain for the last one month | | | | | | |
| --- | --- | --- | --- | --- | --- | --- |
| Q1. | Did you have any pain (including numbness) in your neck or upper limbs (shoulder, arm, or hand)? | | | | | |
|  | 0. No pain | 1. Mild pain | 2. Moderate pain | 3. Considerable pain | | 4. Severe pain |
| Q2. | Did you have any pain in your back lower back or buttocks? | | | | | |
|  | 0. No pain | 1. Mild pain | 2. Moderate pain | 3. Considerable pain | 4. Severe pain | |
| Q3. | Did you have any pain (including numbness) in your lower limbs (hip, thigh, knee, calf, shin, ankle, or foot)? | | | | | |
|  | 0. No pain | 1. Mild pain | 2. Moderate pain | 3. Considerable pain | 4. Severe pain | |
| Q4. | To what extent has it been painful to move your body in daily life? | | | | | |
|  | 0. No pain | 1. Mild pain | 2. Moderate pain | 3. Considerable pain | 4. Severe pain | |
| Following are questions about your usual daily life for the last one month | | | | | | |
| Q5. | To what extent has it been difficult to get up from a bed or lie down? | | | | | |
|  | 0. Not difficult | 1. Mildly difficult | 2. Moderately  difficult | 3. Considerably difficult | 4. Extremely  difficult | |
| Q6. | To what extent has it been difficult to stand up from a chair? | | | | | |
|  | 0. Not difficult | 1. Mildly difficult | 2. Moderately difficult | 3. Considerably difficult | 4. Extremely difficult | |
| Q7. | To what extent has it been difficult to walk inside the house? | | | | | |
|  | 0.Not difficult | 1. Mildly difficult | 2. Moderately difficult | 3. Considerably difficult | 4. Extremely difficult | |
| Q8. | To what extent has it been difficult to put on and take off shirts? | | | | | |
|  | 0.Not difficult | 1. Mildly difficult | 2. Moderately difficult | 3. Considerably difficult | 4. Extremely difficult | |
| Q9. | To what extent has it been difficult to put on and take off trousers and pants? | | | | | |
|  | 0.Not difficult | 1. Mildly difficult | 2. Moderately difficult | 3. Considerably difficult | 4. Extremely difficult | |
| Q10. | To what extent has it been difficult to use the toilet? | | | | | |
|  | 0.Not difficult | 1. Mildly difficult | 2. Moderately difficult | 3. Considerably difficult | 4. Extremely difficult | |

| Q11. | To what extent has it been difficult to wash your body in the bath? | | | | | | |
| --- | --- | --- | --- | --- | --- | --- | --- |
|  | 0. Not difficult | | 1. Mildly difficult | 2. Moderately difficult | 3. Considerably difficult | | 4. Extremely difficult |
| Q12. | To what extent has it been difficult to go up and down stairs? | | | | | | |
|  | 0. Not difficult | 1. Mildly difficult | | 2. Moderately difficult | 3. Considerably difficult | 4. Extremely difficult | |
| Q13. | To what extent has it been difficult to walk briskly? | | | | | | |
|  | 0. Not difficult | 1. Mildly difficult | | 2. Moderately difficult | 3. Considerably difficult | 4. Extremely difficult | |
| Q14. | To what extent has it been difficult to keep yourself neat? | | | | | | |
|  | 0. Not difficult | 1. Mildly difficult | | 2. Moderately difficult | 3. Considerably difficult | 4. Extremely difficult | |
| Q15. | How far can you keep walking without rest? (please select the closet answer) | | | | | | |
|  | 0.  More than 2-3 km | | 1. approximately 1 km | 2. approximately 300 m | 3. approximately 100 m | | 4. approximately 10 m |
| Q16. | To what extent has it been difficult to go out to visit neighbors? | | | | | | |
|  | 0. Not difficult | 1. Mildly difficult | | 2. Moderately difficult | 3. Considerably difficult | 4. Extremely difficult | |
| Q17. | To what extent has it been difficult to carry objects weighing approximately 2 kg (2 standard milk bottles or 2 PET bottles each containing 1 liter)? | | | | | | |
|  | 0. Not difficult | 1. Mildly difficult | | 2. Moderately difficult | 3. Considerably difficult | 4. Extremely difficult | |
| Q18. | To what extent has it been difficult to go out using public transportation? | | | | | | |
|  | 0. Not difficult | 1. Mildly difficult | | 2. Moderately difficult | 3. Considerably difficult | 4. Extremely difficult | |
| Q19. | To what extent have simple tasks and housework (preparing meals, cleaning up, etc.) been difficult? | | | | | | |
|  | 0. Not difficult | 1. Mildly difficult | | 2. Moderately difficult | 3. Considerably difficult | 4. Extremely difficult | |
| Q20. | To what extent have load-bearing tasks and housework (cleaning the yard, carrying heavy bedding, etc.) been difficult? | | | | | | |
|  | 0. Not difficult | 1. Mildly difficult | | 2. Moderately difficult | 3. Considerably difficult | 4. Extremely difficult | |
| Q21. | To what extent has it been difficult to perform sports activity (jogging, swimming, gate ball, dancing, etc)? | | | | | | |
|  | 0. Not difficult | 1. Mildly difficult | | 2. Moderately difficult | 3. Considerably  difficult | 4. Extremely difficult | |
| Q22. | Have you been restricted from meeting your friends? | | | | | | |
|  | 0. Not restricted | 1. Slightly restricted | | 2. Restricted about half the time | 3.Considerably restricted | 4. Gave up all activities | |

| Q23. | Have you been restricted from joining social activities (meeting friends, playing sport, engaging in activities and hobbies, etc.)? | | | | |
| --- | --- | --- | --- | --- | --- |
|  | 0. Not restricted | 1. Slightly restricted | 2. Restricted about half the time | 3.Considerably restricted | 4. Gave up all activities |
| Q24. | Have you ever felt anxious about falls in your house? | | | | |
|  | 0. Have not felt anxious | 1. Have occasionally felt anxious | 2. Have sometimes  felt anxious | 3. Have often felt anxious | 4. Have constantly felt anxious |
| Q25. | Have you ever felt anxious about being unable to walk in the future? | | | | |
|  | 0. Have not felt anxious | 1. Have occasionally felt anxious | 2. Have sometimes  felt anxious | 3. Have often felt anxious | 4. Have constantly felt anxious |
